# Lessons From the COVID-19 Pandemic Response Implementation: A Case Study of South Sudan and Sierra Leone

**DOI:** 10.9745/GHSP-D-23-00180

**Published:** 2024-02-20

**Authors:** Evans Nyasimi Mokaya, Nathan Anyuon Atem, George Awzenio, Lawrence Mukombo, Tom Sesay, Desmond Maada Kangbai, Haurace Nyandemoh, Patience Musanhu

**Affiliations:** a South Sudan Ministry of Health, Juba, South Sudan.; b South Sudan World Health Organization Country Office, Juba, South Sudan.; c Sierra Leone Ministry of Health and Sanitation, Freetown, Sierra Leone.; d Gavi, The Vaccine Alliance, Geneva, Switzerland.

## Abstract

**Introduction:**

The COVID-19 pandemic caused havoc to health systems worldwide and in countries that already had weak health systems. There are lessons to be learned that could contribute to improved response preparedness to future public health emergencies, but there is little documentation on best practices in fragile countries. We describe lessons from South Sudan and Sierra Leone during the COVID-19 response implementation.

**Methods:**

We conducted a retrospective descriptive analysis of COVID-19 vaccination implementation at national and subnational levels between 2020 and 2022 in South Sudan and Sierra Leone to identify those practices that had a positive impact on public health.

**Results:**

Several interventions were identified that not only improved the COVID-19 situation but also had a positive effect on routine immunizations. The development of a near-real-time vaccination dashboard gave stakeholders a quick look at vaccine implementation, allowing them to make decisions based on current data. The experience acquired from deploying the COVID-19 dashboard has since been applied to the development of a routine immunization dashboard in South Sudan. Surge vaccination was an effective approach to improving COVID-19 vaccination uptake. A measles reactive campaign was conducted during the initial stages of the pandemic when movement was restricted; experience gained from that effort was subsequently applied to COVID-19 mass vaccination initiatives and outbreak reactive campaigns. Additional vaccinators recruited for COVID-19 response also received comprehensive Immunization in Practice training, allowing them to provide routine childhood vaccinations alongside COVID-19 vaccination, contributing to the maintenance of routine vaccination services in both countries.

**Conclusion:**

Lessons were learned during the COVID-19 response implementation that have had a positive impact on routine health services. However, it is essential that these effects are maintained and further refined to strengthen the country’s preparedness for future public health emergencies and better support the broader immunization service delivery.

## Abstract (precis)

In South Sudan and Sierra Leone, the implementation of the COVID-19 response generated lessons that could contribute to the improvement of routine health services and preparedness for future public health emergencies.

## Teaser

**Key Findings:**

- During a health crisis, attention should not be completely diverted from other essential services, as this could result in the re-emergence of disease and impede disease elimination and eradication efforts. In this study, integrating childhood immunization into COVID-19 vaccination proved to be an effective recovery strategy that maintained routine immunization coverage during the pandemic.
- The interactive dashboard example highlights the role of technology in boosting immunization services as an easy and cost-effective method of increasing accountability and data-driven decision-making.
- Lessons learned from integrating COVID-19 and routine immunizations during the acute phase of the COVID-19 pandemic served as the basis for integrating COVID-19 vaccination into primary health care services in both countries.

**Key Implications:**

- Program managers in Sierra Leone and South Sudan should apply the lessons learned from the COVID-19 pandemic to improve health care delivery and prepare the countries for future public emergencies.
- Policymakers and supporting agencies in South Sudan and Sierra Leone should advocate for increased health sector funding to maintain COVID-19 response innovations and health care personnel.

Plain language article summary available.

## INTRODUCTION

The COVID-19 pandemic has been described as a “once-in-a-lifetime” pandemic that unexpectedly wreaked havoc on all countries, regardless of wealth.^1^ Nonetheless, the pandemic has been particularly challenging for low- and middle-income countries, especially already fragile countries like South Sudan and Sierra Leone.

Years of conflict and low government investment in health have left the health systems in South Sudan and Sierra Leone weak to effectively handle a pandemic like COVID-19. The health systems of South Sudan and Sierra Leone already lacked human health resources, pharmaceutical and other medical supplies, and infrastructure even before the first COVID-19 case was reported.^2^ Additionally, South Sudan was facing the effects of severe flooding, disease outbreaks, economic collapse, and a political transition.^3^^,^^4^ The health care systems of both countries were further strained by the pandemic and subsequent preventive measures. Existing health care personnel could not adequately respond to the pandemic while also providing routine services. Donors and humanitarian agencies were caught unprepared and had to balance the COVID-19 response and routine health service delivery. Drugs, vaccinations, and other important health supplies were delayed due to the partial lockdown in Juba, South Sudan.^3^^,^^5^

Based on disease outbreak patterns over the previous decade and a rapidly globalizing society, more pandemics should be expected. Outbreaks occurring in the past decade of Ebola, H1N1 influenza virus, Zika virus, and now COVID-19 indicate a worrisome pattern.^6^ Therefore, it is important for all relevant key stakeholders to reflect on past and current outbreaks and prepare for potential future pandemics. Countries that experienced severe acute respiratory syndrome and other significant illness outbreaks applied the lessons to construct more robust and emergency-ready health systems.^5^^,^^7^^–^^9^

As the COVID-19 pandemic continues to evolve, countries continue to learn from their experiences. Countries with adequate surge capacity—the ability to quickly mobilize adequate numbers of health workers—responded better to the pandemic. Decision-makers in countries with integrated disease surveillance and health information systems were able to use real-time data to track vaccination uptake, early disease identification, disease spread, and essential service access and use during the pandemic, enabling them to make timely and better-informed decisions regarding vaccinations and prevention strategies.^10^^–^^12^

South Sudan and Sierra Leone’s COVID-19 pandemic response implementation (2020–2022) provides complementary learning opportunities for nations with similar contexts. These experiences helped both countries maintain childhood immunization coverage while scaling up COVID-19 vaccination. The COVID-19 pandemic contributed to the training of health care workers countries may rely on to respond to future health crises. In addition, the countries are also using the COVID-19 data management lessons to improve routine immunization data management. Importantly, these lessons provided a foundation for integrating COVID-19 vaccination into primary health care after the end of the emergency phase of the disease response. This article explores the experiences of South Sudan and Sierra Leone together due to the similar characteristics in the fragility of their health systems in the post-conflict period.

South Sudan and Sierra Leone’s COVID-19 pandemic response implementation provides complementary learning opportunities for nations with similar contexts.

## METHODS

This article presents a retrospective, longitudinal study of the experiences of Sierra Leone and South Sudan during the COVID-19 pandemic. The results are based on a descriptive analysis of data and findings collected on the implementation of the COVID-19 vaccination in Sierra Leone and South Sudan during 2020–2022. We reviewed official Ministry of Health (MOH) documents (2 intra-action review reports, the National Deployment and Vaccination Plan, and quarterly COVID-19 deployment progress reports) and analyzed the COVID-19 and routine immunization data on the COVID-19 deployment dashboard and the DHIS2.

The experiences presented in this article are part of a range of responses to the COVID-19 pandemic that have already been implemented in South Sudan and Sierra Leone. These examples come from all levels of health care delivery and involve MOH colleagues and partner staff supporting the Essential Program on Immunization (EPI) in both countries. As the deployment of the COVID-19 vaccination was conducted within the existing structures of EPI and with the assistance of in-country technical partners, the strategies and time frames presented vary by each country.

### Study Settings

#### South Sudan

A protracted civil war that ultimately led to the independence of South Sudan in 2011 severely degraded the health system. After independence, the health system remained weak, resulting in continued poor health outcomes. South Sudan experienced its own civil war from 2013 to 2020, which brought new challenges, such as insufficient government funding for health, demotivated health workers due to delayed and unpredictable remuneration, and limited capacity to manage the health system. Although service delivery improved at times, especially during periods of relative peace (2006–2013 and 2020–2022), service delivery and health systems remained inadequate and continue to be driven mostly by development partners. Ongoing challenges include the continuing deterioration of health infrastructure, a substantial rural population in difficult-to-reach locations, recurrent intercommunal conflict, and significant seasonal flooding. The current health sector funding is primarily provided by the Health Pool Fund and The World Bank. The EPI in South Sudan relies significantly on donor assistance, as the availability of local resources is low, which presents a barrier to the long-term sustainability of immunization services.

Before the outbreak of COVID-19, South Sudan’s capacity to respond rapidly to public health emergencies through immunizations as measured by the Joint External Evaluation indicators^13^ was inadequate. The country scored 2 (limited capacity), 3 (developed capacity), and 3 (developed capacity) for measles coverage, national vaccine access and delivery, and mass vaccination for vaccine-preventable disease epidemics, respectively. This represents an improvement over the 2017 evaluation.^14^ According to the World Health Organization (WHO)/UNICEF Estimates of National Immunization Coverage, South Sudan had a 65% measles coverage rate at the national level.^15^ However, the national average coverage conceals disparities at the subnational level and unequal access to immunization services. The country’s EPI comprehensive and costed multiyear plan (2018–2022) aimed to achieve a measles coverage rate of 80% by 2020.^16^ The country’s 2020 annual operational plan elaborated on specific activities, including a national measles supplemental activity to boost the community immunity profile against measles. The immunization data reporting via the DHIS2 system was lacking in completeness and timeliness, and despite the inclusion of data quality procedures in the plan, their execution was irregular.

All 80 counties in South Sudan had reliable cold chain capacity adequate for routine vaccination and supplemental immunization activities.^17^ Moreover, of 1,400 health facilities that offered immunization services in 2019, 857 had a functioning cold chain. However, the distribution of health facilities and, by extension, the cold chain is not equitable, which restricts access to the cold chain in the country’s disadvantaged regions. According to the 2019 Joint Appraisal report, monitoring of vaccine stock levels is limited to the national and state levels.^18^ The report highlights that no vaccine stock-outs were reported at the national and state levels. Nevertheless, the report acknowledged the existence of anecdotal reports regarding stock shortages of bacille Calmette-Guerin and inactivated polio vaccines at health facilities.

South Sudan had experience with the experimental Ebola virus vaccine as well as multiple national vaccination efforts for measles, polio, and meningitis.^19^ The national vaccination campaigns targeted all populations, including the marginalized and vulnerable populations, as directed by the comprehensive bottom-up microplanning process. The national drug and food control authority had a fast-tracked approval process for new pharmaceuticals that were WHO-prequalified, including emergency approval for the use of experimental vaccines in epidemics of new pathogens.^20^ New and experimental vaccine coverage and safety monitoring were conducted daily using the ODK dashboard supported by WHO. However, the adverse events following immunization (AEFI) monitoring system was weak.^18^

#### Sierra Leone

Between 1991 and 2002, Sierra Leone’s health system development was hindered by a protracted civil war. The Ebola outbreak in 2014 killed approximately 7% of the health care workforce and exacerbated a decline in the effectiveness of the country’s health care system.^2^ Shortages of doctors, nurses, and midwives continue to be widespread. The lack of doctors is worsened by their preference to work in urban regions. Because of these obstacles, the country has some of the poorest health statistics in the world.

Before the COVID-19 outbreak, Sierra Leone’s readiness to address public health emergencies through immunization, as measured by the Joint External Evaluation indicators,^13^ was at level 3 (developed capacity) for measles coverage, national vaccine access and delivery, and mass vaccination for vaccine-preventable disease epidemics. According to WHO/UNICEF Estimates of National Immunization Coverage, the national coverage for measles in Sierra Leone was 93%.^21^ The country’s EPI costed multiyear plan (2017–2021) aimed to maintain the high measles coverage rate of 94% by 2020.^22^

According to the Joint Appraisal report, all districts in Sierra Leone, except for 2 new districts established in 2019, had adequate cold chain for routine vaccination and supplementary immunization activities.^23^ However, the report highlights that adequate monitoring of vaccine stock levels is limited to the national level, with insufficient stock reporting from districts and limited visibility into vaccine utilization at health facilities. The report highlights that no vaccine stock-outs were reported at the national and district levels.

Sierra Leone had experience with the experimental Ebola virus vaccine in addition to multiple national vaccination campaigns for vaccine-preventable diseases like measles and polio that reached all target populations, including marginalized and disadvantaged populations.^5^ The national drug and food control authority had an expedited approval process for new pharmaceuticals that were prequalified by WHO, as well as emergency approval for the use of experimental vaccines in outbreaks of new pathogens. New and experimental vaccine coverage and safety monitoring were conducted daily using the DHIS2. Although AEFI surveillance had improved, it remained subpar.^23^

## COVID-19 DISEASE AND VACCINATION SITUATION IN SOUTH SUDAN

South Sudan reported its first COVID-19 cases on April 4, 2020. Three waves hit South Sudan: May–July 2020, January–April 2021, and December 2021–January 2022. As of January 2023, 18,142 cases had led to 139 deaths (0.76%) in South Sudan. However, due to limited testing and monitoring capacities, the real number of cases and deaths may be much higher.

In April 2021, South Sudan began administering the COVID-19 vaccine. Since then, the services have expanded to all 10 states and 2 administrative areas in South Sudan. By June 30, 2021, 67.73% (4,146,163) of the target population (adults aged 18 years and older) in South Sudan were fully vaccinated, with 51.50% of the clients being women. In South Sudan, 85.02% of health workers and 80.14% of the elderly had been vaccinated.

### Lessons Learned in South Sudan

Our review of official MOH reports and COVID-19 and routine childhood vaccination data identified 5 lessons learned from the COVID-19 pandemic response in South Sudan related to capacity-building initiatives; recruiting, training, and deploying an expanded workforce to achieve COVID-19 vaccination objectives without hurting routine vaccination coverage; leveraging mobile and outreach approaches to improve COVID-19 and overall vaccine coverage; developing a near-real-time data collection, visualization, and dissemination dashboard to support evidence-based decision-making; and maintaining the ability to mount necessary responses to other public health emergencies by ensuring appropriate use of COVID-19 precautions by health care workers.

#### Building Capacity of EPI Before the COVID-19 Pandemic

Two preexisting initiatives contributed to the alleviation of the shortage of health care staff for the EPI in South Sudan. First, using Gavi, The Vaccine Alliance’s health systems strengthening financing opportunity for fragile countries (Fragility, Emergency, and Refugees), the country recruited 1,488 new vaccinators in the fourth quarter of 2019 and first quarter of 2020.^24^^,^^25^ Second, as one of the polio end-game legacy initiatives in South Sudan, the U.S. Centers for Disease Control and Prevention in collaboration with the African Field Epidemiology Network, began supporting the MOH’s training of 56 midlevel EPI officers in 2015 using a competency-based and field-based approach.^26^ By 2020, all the officers in training had graduated and were working with the MOH. These 2 EPI capacity-building initiatives contributed greatly to the COVID-19 pandemic response. The Gavi-supported MOH vaccinators were instrumental in maintaining routine vaccinations, while the state-based officers trained by the U.S. Centers for Disease Control and Prevention and African Field Epidemiology Network supervised EPI services in 2020 when the national-level supervisors could not go to subnational levels because of the partial lockdown. Of note, even during the lockdown period, supportive supervision and Immunization in Practice trainings for the newly recruited vaccinators continued under the supervision of the state-based officers (the Immunization in Practice trainings were simplified and adapted for use at the subnational level).

#### Recruiting, Training, and Deploying COVID-19 Vaccinators While Maintaining Regular Vaccination Coverage

Based on the lessons learned from the 2019 Ebola vaccine deployment, the MOH guidance stipulated that only qualified nurses and doctors could give the COVID-19 vaccines. However, due to a preexisting shortage of clinicians, their redeployment to COVID-19 duties caused shortages in other crucial areas of the health care system. Three months after the launch of COVID-19 vaccination in South Sudan, the negative effects of reassigning nurses from routine clinical services to COVID-19 vaccination were clear. For example, malaria cases were reported to have soared, but there were few nurses to attend to the patients, resulting in delayed management of the cases.

To achieve high and equitable vaccination coverage without jeopardizing existing health care services, the leadership of the MOH revised the policy and recommended the hiring of 1 nurse to work alongside 2 experienced non-nurse vaccinators. A total of 570 additional health care providers were hired in South Sudan’s 80 counties. A majority (71%) of the additional nurse and non-nurse vaccinators received the same 5-day Immunization in Practice training as the regular vaccination staff. The training prepared the additional human resources to provide routine childhood vaccination and COVID-19 vaccination. These additional human resources conducted multiple rounds of targeted and integrated mobile and outreach sessions, contributing to the reported increase in coverage for both routine childhood vaccination and COVID-19 vaccination. As was envisaged, the non-nurse vaccinators continued to learn on the job from the nurse vaccinators, leading to improved quality in both COVID-19 and routine vaccination. This approach improved the conditions for ensuring equity and effectiveness by overcoming key human resource constraints. Currently, the COVID-19 vaccination is administered by 2 nonclinical vaccinators who have historically administered childhood vaccinations, allowing the nurses to concentrate on other clinical responsibilities.

Recruiting, training, and deploying additional vaccinators to conduct multiple rounds of vaccine sessions contributed to the reported increase in coverage for both routine childhood vaccination and COVID-19 vaccination.

#### Using COVID-19 Vaccination Outreach and Mobile Clinics to Provide Routine Childhood Vaccines

Eastern Equatoria State’s first dose of pentavalent vaccine (penta 1) coverage was suboptimal in 2019 and 2020 (40% and 48%, respectively). Low access to vaccination services was a driver for the state’s suboptimal performance. To reverse the trend, the state’s leadership and its supporting partners made an informed decision to begin integrating routine childhood vaccination into the outreach and mobile services for COVID-19 vaccination. The integrated outreach and mobile services were launched in the fourth quarter of 2021, coinciding with the start of South Sudan’s dry season and the stabilization of COVID-19 vaccine supplies. To actualize this plan, the state recruited an additional 202 mobile and outreach-focused vaccine providers. These vaccinators were trained using the Immunization in Practice curriculum^27^ in addition to training specific to COVID-19 vaccines, making them qualified to provide routine childhood vaccination alongside the COVID-19 vaccines.

Integrating childhood vaccination alongside COVID-19 mobile and outreach programs required meticulous preparation. Community mobilizers notified the community in advance that both services would be offered and allayed anxieties regarding COVID-19 immunizations for children. To prevent vaccine mix-ups, vaccinations were appropriately labeled and transported in separate vaccine carriers, and the immunization sites were set up separately for children and adults. However, after screening, clients were cross-referred between the immunization sites.

**FIGURE 1:**
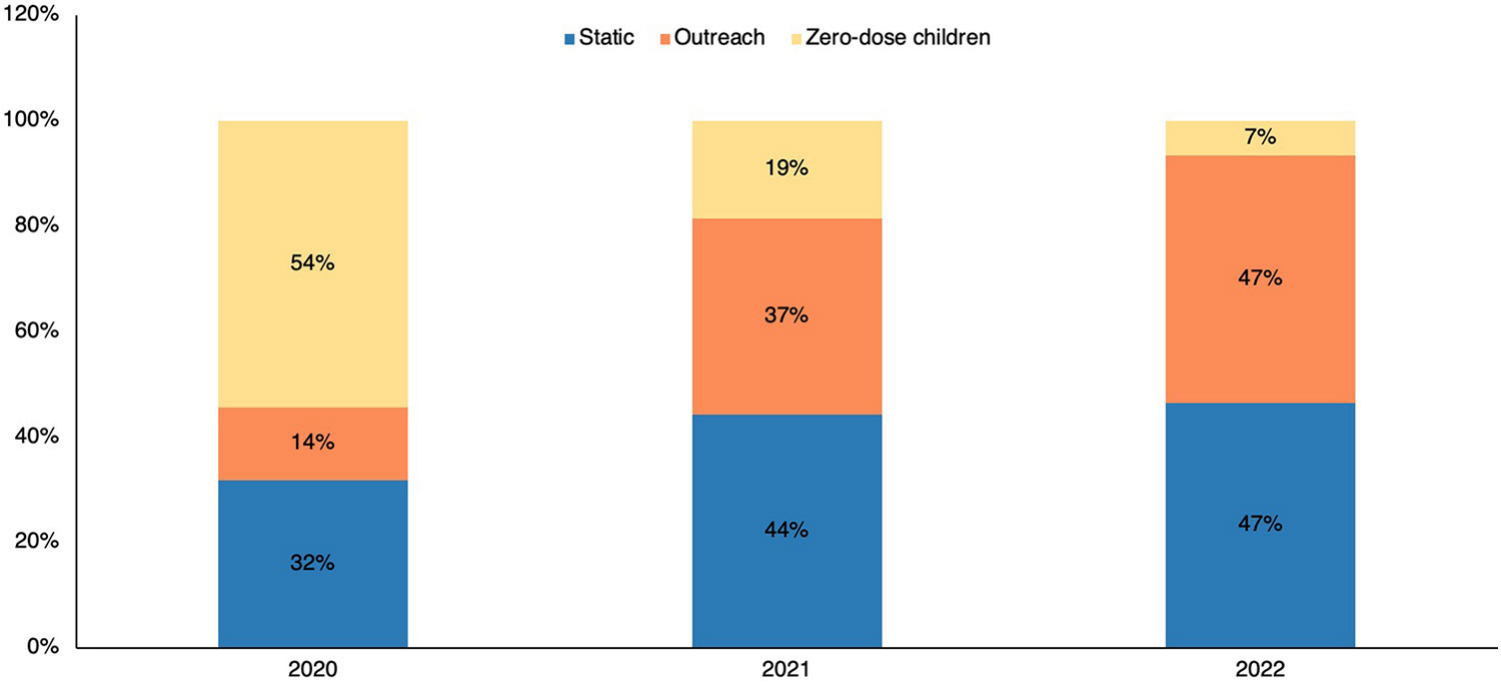
Children Receiving First Dose of the Pentavalent Vaccine by Delivery Strategy and Zero-Dose Children, Eastern Equatoria State, South Sudan, January–September, 2020–2022 Source: DHIS2 South Sudan.

Figure 1 depicts an increased uptake of penta 1 and a reduction in the number of zero-dose children in the first 2 quarters of 2021 and 2022 compared to the same period in 2020. The graph further shows the relative contribution of static and outreach and mobile services to overall vaccine uptake between 2020 and 2022; the contribution of outreach and mobile vaccination services increased from 14% in 2020 to 37% and 47% in 2021 and 2022, respectively.

In 2023, 5 more states integrated COVID-19 with regular immunization using learning from the pilot phase in the equatorial region. Through scale-up, the country learned the following.

- For the integration to be successful, additional vaccinators were required, particularly in institutions where vaccinators had to be assigned to special clinics for patients with comorbidities.
- Vaccinators were reluctant to administer COVID-19 vaccinations in static facilities because they had become used to the campaign-based administration of COVID-19 vaccination. However, they were willing to administer the vaccine during outreach and mobile services.
- Adults, particularly males, were less inclined to wait in line for the COVID-19 vaccination beside the majority of female caretakers bringing their children for childhood vaccinations.
- Some caretakers stayed away from the integrated sessions for fear of inadvertently administering the COVID-19 vaccine to a child, especially when the same team served both adults and children.

During the first half of 2023, integration resulted in increased uptake of routine immunization services, particularly in the most remote areas accessible via mobile services. In contrast to the campaign method, the uptake of COVID-19 vaccines through regular services was low, particularly in static facilities. This was partially attributable to the community’s getting used to receiving services closest to their residences, which resulted in a reluctance to seek services in static facilities.

After it was determined that COVID-19 disease was not a public health emergency of international concern, the country embarked on addressing some challenges from the previous phased implementation to ensure seamless integration of COVID-19 with routine immunization and other primary health care interventions. In particular, the extra vaccinators were kept to help with both routine immunization and COVID-19 immunization. The training of the vaccinators had already been integrated. The vaccinators were made aware of the need to accept COVID-19 as an additional regular vaccine. Campaigns were switched to mobile and outreach services so that the elderly population could still be reached close to their homes.

#### Using a Dashboard to Visualize COVID-19 Vaccine Uptake and Safety Surveillance Data

South Sudan initiated near-real-time monitoring of vaccine uptake and AEFI surveillance for COVID-19 vaccinations at the outset of the vaccination initiative using a dashboard built with Power BI software (https://tinyurl.com/bvyutz8j). This was done to facilitate the extraction and visualization of COVID-19 vaccine administration and safety data by MOH officers and other COVID-19 vaccination stakeholders.

The COVID-19 Vaccine Dashboard was developed to aggregate and visualize summary coverage and safety data from all 80 counties in the country. The dashboard monitored critical COVID-19 vaccination indicators to enable a rapid daily evaluation of the COVID-19 vaccination situation. The aggregated vaccination data were shared daily by trained health workers from each immunization site using the ODK application installed on Android smartphones. The national MOH officers and partners were given viewer access to observe and engage with the dashboard, but they could not change the data. The vaccinators and their supervisors were given data input rights. The data team, led by the lead consultant, had authorization to clean all dashboard data.

**FIGURE 2:**
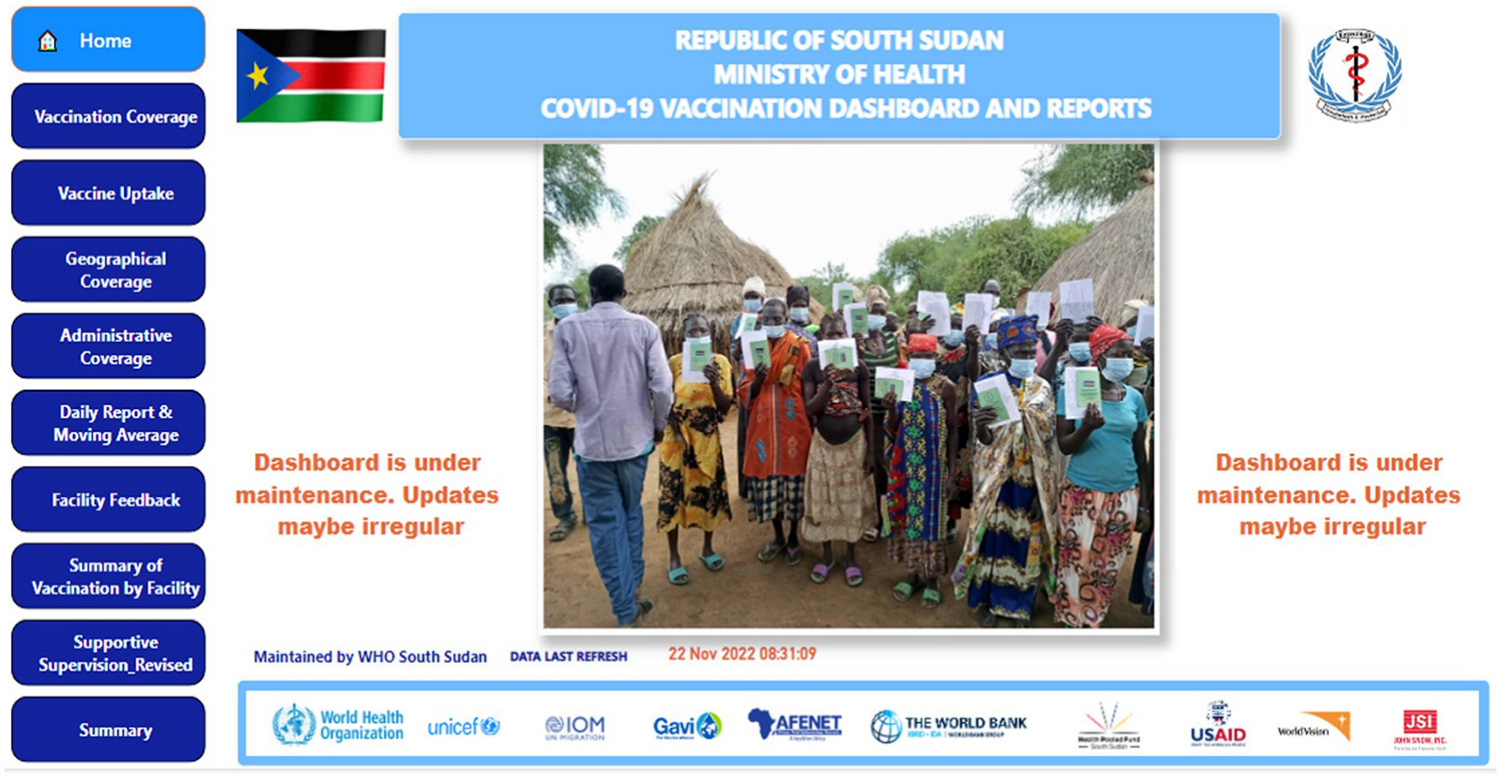
COVID-19 Vaccination Dashboard Home Page, South Sudan, 2023

The dashboard included indicators for 9 important sections: vaccine coverage, vaccination uptake, geographic coverage, administrative coverage, a daily report and moving average, facility feedback, a summary of immunizations by facility, supportive supervision, and a summary (Figure 2). Each section’s relevant indicators were defined based on end users’ needs. The indicators in a category were used to construct visualizations, which were then integrated into single-page dashboards; each dashboard comprises at least 1 visualization. There are currently 9 different single-page reports. To aid in the speedy review of data, various visualization methods were used (e.g., bar charts, histograms, heat maps, and line charts). To assist users in quickly accessing the single-page report, a navigation panel with section headers was created on the dashboard’s left side.

Dashboard features allowed users to swiftly evaluate and share dashboard visualizations. The single-page dashboards comprised both interactive and static visuals. Viewers could drill down to individual data views using filters by selecting parameters of interest such as time frame, demographics, COVID-19 vaccination type, location, implementing partner, and dose numbers. Viewers could take screenshots of both interactive and static dashboards to use in presentations and quickly share with others. The dashboard visualizations were evaluated daily by the vaccine safety teams for AEFI monitoring and weekly during COVID-19 Technical Working Group sessions to inform decisions. The dashboard’s visualizations have been used to inform policymakers’ decision-making. For example, dashboard data were used to determine priority counties for the Intensified COVID-19 Vaccination Optimization. Select dashboard visualizations were used in weekly presentations to the COVID Steering Committee, donor meetings, the Inter-Agency Coordination Committee, and South Sudan Immunization Technical Advisory Group meetings to provide information on the status of COVID-19 immunization coverage by specific populations, sex, vaccine safety, and vaccine consumption.

#### Conducting Measles Campaigns During the COVID-19 Pandemic and Flooding Seasons

Aweil East County reported a measles outbreak in the first quarter of 2020. An outbreak-reactive measles vaccination campaign was conducted in February 2020 but did not stop the outbreak, which ultimately resulted in 8 measles-related deaths in April and May 2020. The first case of COVID-19 was recorded in South Sudan in April, and as a result, movement and gatherings were restricted throughout the country, making it difficult to respond to the developing measles outbreak in Aweil East County. In June 2020, a special meeting of the EPI technical working group recommended conducting another outbreak-reactive vaccination campaign in Aweil East under strict observance of the COVID-19 preventive measures. The Table lists the specific precautions made to facilitate the implementation of the campaign in the context of the COVID-19 pandemic.

**TABLE.** Precautions to Facilitate the Implementation of the Measles Campaign in the Context of COVID-19 Pandemic in South Sudan

**Thematic Area**	**Specific Amendment in the Context of COVID-19 Disease**
Microplanning	The microplanning templates were modified to include provision of the necessary resources for the prevention and control of COVID-19 infections.
	The duration of the campaign was extended from 7 to 10 days.
	The anticipated average number of children to be vaccinated per team each day was reduced from 100 to 70.
Training	The measles supplemental immunization activity training materials were updated to include information on COVID-19 prevention.
	A combination of virtual and in-person training sessions were used to provide cascade training for national and subnational trainers, supervisors, and health care workers.
	In-person trainings were conducted with fewer participants (10–20 participants) and the appropriate safeguards, including face masks, hand sanitizer, and physical distancing. Practical sessions on preventing COVID-19 transmission at the site of service delivery were done.
Service delivery	Fixed and temporary static vaccination sites were established in open areas with sufficient ventilation and enough space to allow physical distance while queuing for services.
	To bring services closer to the community, more outreach and mobile services were conducted in temporary posts in the villages.
Infection prevention	Each member of the immunization team received enough masks and hand sanitizers to use 1 each day for 10 days. All health personnel and volunteers were encouraged to wear face masks, and those administering immunizations were urged to disinfect their hands between clients.
	Clients were encouraged to wear masks, including improvised cloth masks when proper masks were not available. Volunteers encouraged clients to sanitize or wash their hands at vaccination sites that had handwashing facilities.
	At each vaccination site, clients were screened for high temperatures using infrared thermometers. In vaccination sites where there was no infrared thermometer, health care workers screened individuals by asking for symptoms consistent with COVID-19, such as fever, cough, and/or difficulties in breathing.

Eighty-four percent (89,425) of children aged 6–59 months received the measles vaccine. The campaign (together with the 2 preceding measles-reactive immunization efforts) was successful in containing the outbreak, as no cases were reported in July 2020 or later months of 2020. Notably, there was no evidence of an increase in COVID-19 disease transmission in the county that could be attributable to the campaign.

## COVID-19 DISEASE AND VACCINATION SITUATION IN SIERRA LEONE

On March 30, 2020, Sierra Leone reported its first COVID-19 cases. As of January 2023, there were 7,757 confirmed cases and 125 fatalities in the country. Due to limited testing, the actual number of cases and fatalities could be significantly higher.

Sierra Leone began administering the COVID-19 vaccine in April 2021. Since then, the services have expanded to each of Sierra Leone’s 16 districts. By June 30, 2023, 73.2% (3,644,025) of the adults and adolescents were fully vaccinated, and 11% of adults and adolescents had received booster dosage. Of note, 47% of health workers and 33% of the elderly were vaccinated.

### Lessons Learned in Sierra Leone

Our review of official MOH reports and COVID-19 and routine childhood vaccination data revealed 4 lessons learned from the response to the COVID-19 outbreak in Sierra Leone relating the use of political leaders to address myths and misconceptions, the use of a campaign strategy to increase the uptake of COVID-19 vaccination, the use of COVID-19 mobile and outreach sessions to maintain regular childhood vaccine coverage, and the use of the COVID DHIS2 module to reduce COVID-19 vaccination defaulter rates.

#### Leveraging Political Leadership to Address Myths and Misconceptions

At the start of the COVID-19 vaccination, there were widespread misconceptions about the vaccines, including numerous claims that COVID-19 vaccines caused serious illness, death, or disability among vaccine recipients. This misinformation contributed to an initial hesitancy among the public to obtain the vaccine. To overcome this challenge, a decision was made to commence the vaccination process with vaccination of the President, cabinet ministers, and parliamentarians on March 15 during a well-publicized event at the State House. Pictures and videos were widely shared on social and mainstream media across the country. This helped community members gain confidence in the vaccination process, dispel rumors that the vaccine was harmful, and encourage vaccine uptake.

#### Using a Surge Vaccination Campaign

**FIGURE 3:**
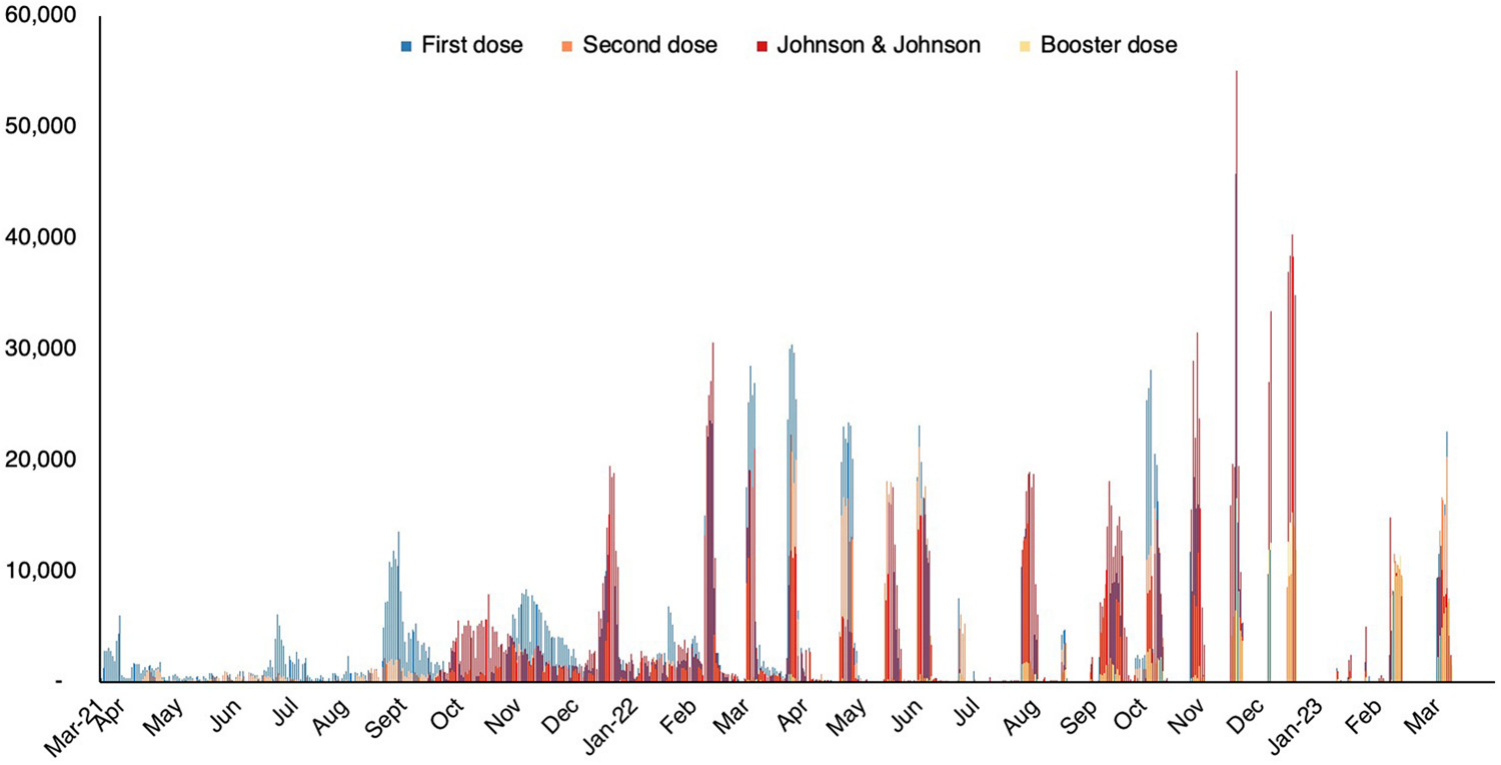
COVID-19 Vaccination Uptake by Vaccine Type and Dose Series, Sierra Leone,^a^ 2021–2023 ^a^ Peaks correspond to surge vaccination exercises. Sierra Leone implemented 18 rounds of surge vaccination as depicted by the peaks in the graph. Source: DHIS2 Sierra Leone.

An initial vaccination strategy using 38 static and 34 outreach vaccination teams resulted in low COVID-19 vaccine coverage. Even after increasing the number of static and outreach teams to 1,300, coverage remained suboptimum. This was mostly because clients rarely visited the facilities, reportedly because of low perceived risk of contracting COVID-19 infection. To accelerate uptake, the country adopted surge vaccination as an additional approach to routine vaccination. Leveraging lessons learned from previous vaccine intensification campaigns for measles and polio, the surge vaccination campaign involved deploying an increased number of vaccination teams targeting high-volume sites such as markets and universities in a 7-day campaign. There was intense community mobilization using the radio and television, as well as community-to-community household engagement by the community health workers starting in advance of the surge vaccination exercises. These intensive monthly vaccinations lead to high vaccination coverage (Figure 3).

#### Integrating COVID-19 Vaccination With Routine Vaccination

**FIGURE 4:**
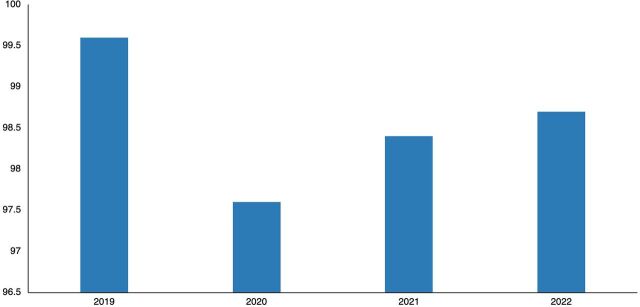
Third Dose Pentavalent Vaccination Coverage, Sierra Leone, 2019–2022 Source: DHIS2, Sierra Leone.

In 2020, the immunization rate in Sierra Leone decreased due to the COVID-19 pandemic. The integration of routine immunization with the COVID-19 vaccination allowed for recovery and maintenance of coverage in 2021 and 2022 (Figure 4). The COVID-19 vaccination teams, which consisted of 2 vaccinators, 1 social mobilizer, and 1 data entry clerk, administered routine vaccinations along with the COVID-19 vaccines during the regular COVID-19 vaccination and surge sessions. There were also additional efforts undertaken to identify children who had missed routine immunizations and provide them with catch-up doses.

The integration of routine immunization with the COVID-19 vaccination allowed for recovery and maintenance of vaccine coverage in 2021 and 2022 in Sierra Leone.

After the emergency phase of COVID-19 was declared over, COVID-19 vaccination was integrated with routine immunization services. Clients in need of boosters or primary series can access the vaccine in either the static or outreach vaccination services. Persons with comorbidities are encouraged to access the vaccines from the vaccination centers.

#### Using a Case-Based Immunization Database

To collect COVID-19 vaccination data, a case-based vaccination database was built within the DHIS2 platform with adequate data protection systems. Vaccination teams were given tablets to use at strategically situated health facilities that served as data entry hubs. The case-based data system ensured that all relevant information about the vaccine recipients was captured in the vaccine database. The system was designed to deliver congratulatory messages to vaccine recipients, remind clients of their next dose, and identify and follow up with defaulters. For the latter, repeated messages would be sent to defaulting clients automatically on a weekly basis until they received their second dose. This contributed to the attainment of high coverage of fully vaccinated clients. The use of the case-based vaccination database for routine immunization is currently being explored.

## DISCUSSION

Despite the fragility of their health systems, South Sudan and Sierra Leone began immunizing against COVID-19 at the same time as other African countries. Although COVID-19 disease affected the uptake of routine immunization services in the second and third quarters of 2020, the reduction was minimal, and recovery was achieved by year’s end.^17^

An overarching lesson from past public health crises is that the effectiveness of a pandemic response largely depends on the resilience of the health system. According to Kruk et al.,^28^ a resilient health system is one in which the health actors, institutions, and populations have the capacity to prepare for and effectively respond to crises, retain basic operations throughout a crisis, and reorganize, if necessary, based on lessons learned during the crisis. Hanefeld et al.^29^ suggest that 3 parameters (workforce issues, health information systems, and health care financing) and 2 cross-cutting ones (governance and values) can be used to assess a health system’s resilience.

Building a resilient health system is not a static or single occurrence or event but rather a series of adaptive measures based on willingness to use past experiences to safeguard life and provide positive health outcomes throughout crises and routine health services.^28^ To do this, the countries must undertake evaluations of health system capacities and weaknesses in advance of crises, invest in improving vulnerable components, provide workforce reinforcements during an emergency, and conduct performance reviews following a crisis.

Before 2020, there was a severe shortage of vaccinators in South Sudan.^24^^,^^30^ Similarly, Sierra Leone had a shortage of medical workers before the COVID-19 pandemic. In both countries, the COVID-19 pandemic exacerbated the workforce shortage. The most effective means of sustaining routine services while deploying COVID-19 vaccinations was to recruit more vaccinators. Options available to other countries to address the time-limited heightened demand for health care workers during a pandemic, such as reengaging retired health care workers, reassigning existing staff, and hiring redundant workers,^31^ are limited in fragile countries. For example, unlike in other countries with retired health care workers, the lack of a clear pension scheme in South Sudan has motivated health staff to continue working for as long as possible, even after reaching the official retirement age. Collaboration with the private sector is critical to meeting the surge in health care demand during a pandemic, but the public-private relationship needs to have been established and policies put in place long before a pandemic, especially on the roles and the terms of engagement.^32^ A clear policy on public-private partnership in the provision of health services during health emergencies is nonexistent in both South Sudan and Sierra Leone. Although international organizations’ ability to deploy health care staff during emergencies is critical, this option is constrained by competition for this limited resource across countries during a pandemic, with priority often going to countries with the highest disease burden. Furthermore, external support is primarily targeted at gap-filling rather than skills transfer; therefore, it contributes less to building a sustainable health workforce.

As the emergency phase of the COVID-19 pandemic winds down, it is imperative that countries leverage existing health platforms to deliver COVID-19 vaccines to the general population to increase the efficiency and sustainability of the COVID-19 vaccination. However, in low- and middle-income countries, there are few studies on integrating COVID-19 and regular childhood immunizations. Moreover, South Sudan and Sierra Leone lacked experience in vaccinating adults before the COVID-19 pandemic, as the routine services focused on childhood vaccination. The integration of COVID-19 vaccinations for adults and adolescents into routine services was a new area for both countries.

Although complete integration has not yet been achieved in the 2 countries, significant progress has been made because of the application of successes and addressing the challenges during the phased implementation of COVID-19 and routine immunization services integration. As demonstrated by both countries, in fragile settings with communities that have difficult or limited access to both humanitarian aid and routine services, the enhanced resources mobilized for COVID-19 immunization provided potential routes to better deploy routine vaccinations for children and women, even in hard-to-reach areas.

Integration of COVID-19 and routine childhood vaccination led to more children receiving routine vaccines in South Sudan and Sierra Leone. However, integration was not without challenges. An unpublished report of many vaccine refusals during a Maternal and Neonatal Tetanus Elimination campaign in South Sudan during the COVID-19 pandemic because people believed that the vaccine being used also contained the COVID-19 vaccine was a red flag for the integration.^33^ Anticipating this challenge, the vaccination teams engaged the community and allayed their fears through clear messaging and meticulous implementation to mitigate possible childhood vaccination refusals. The observation that more people were vaccinated in outreach and mobile services compared to static facilities could be attributed to the dependency created by the numerous campaigns. From the perspective of most vaccinators, COVID-19 remains a special service that is delivered either through campaigns or mobile services, which are often linked with intensive community mobilization and additional incentives for the vaccinators. Further, the community, especially the elderly, feels less motivated to seek COVID-19 vaccination in static facilities, expecting the health workers to take the services closer to their homes, as was the norm in the acute phase of the pandemic through campaigns. Another factor impacting static services is the reluctance of adults, especially men, to line up beside the caregivers of children to receive the COVID-19 vaccines.

Important lessons that are driving the integration agenda in both countries include building community trust in health facility services, including vaccination services; alleviating shortages of vaccinators; integrating training packages, including recording and reporting tools; integrating supportive supervision tools; integrating vaccine transportation, including the last mile delivery; and making vaccinators understand the shift from campaigns to routine services after the proclamation of the end of COVID-19 as a public health emergency.

Important lessons that are driving the integration agenda in both countries include building community trust in health facility services, alleviating shortages of vaccinators, and integrating training packages and supportive supervision tools.

Evidence shows that during pandemics, although the focus might shift to the crisis at hand, other public health priorities remain important.^34^^,^^35^ The importance assigned to preexisting or emerging priorities may increase depending on the magnitude and severity of the problem as perceived by the community and its leadership. Faced with a raging measles outbreak, South Sudan became among the first countries to approve a reactive campaign with strict adherence to the specific COVID-19 preventive measures. During the initial stages of the COVID-19 pandemic, several countries that needed to conduct vaccination campaigns in response to disease outbreaks or scheduled preventive measures suspended or delayed them.^36^^,^^37^ The decision was informed by the concerns that the campaigns could propagate the spread of the virus and that the population would not respond appropriately because of the fear of contracting the disease at the vaccination centers.^38^^,^^39^ South Sudan’s experience demonstrated that it is possible to respond to an equally critical priority during a pandemic if sufficient planning and adaptive measures are used. Similar observations were made in Ethiopia and through modeling.^40^^,^^41^

The deployment of the COVID-19 vaccine uptake dashboard in South Sudan was lauded as a game changer because it provided stakeholders with a quick view of vaccine-related data and enabled them to respond to critical questions faster. The dashboard allowed for granular exploration of the data by regions, gender, age groups, deployment strategy, supporting agency, and special populations, which increased accountability and brought to the fore issues of equitable access and uptake of the COVID-19 vaccines. Although the dashboard is innovative, it is not without concerns because of its limitations. Of concern was that the dashboard lacked COVID-19 disease occurrence data, prohibiting users from simultaneously tracking local disease spread and correlating it with vaccine coverage. Also, the dashboard database did not have client-specific information that could be used for follow-up, as was the case in Sierra Leone. In both countries, the lessons learned are being applied to the management of routine immunization data. South Sudan is building an EPI routine immunization dashboard based on the lessons learned from the COVID-19 vaccination dashboard. The dashboard will be improved to include the program inputs, processes, and outputs by agency as a way of improving accountability and encouraging the use of EPI data for decision-making.

Diverse donors supported the interventions and innovations as part of the COVID-19 pandemic response. We recommend that the governments sustain the innovations beyond the period for which they will be funded by donors. Notably, only the maintenance of human resources could be affected if the governments do not assume responsibility for financing additional human resources. Other innovations have already been incorporated into both countries’ EPI.

### Limitations

In this article, pre- and post-analysis are presented as evidence for best practices. Because the interventions were not conceived and implemented as a study from the outset, it is not possible to identify causal pathways. The interventions were implemented in an emergency context. We are aware that other factors may have contributed or acted as confounders to the observations we present. Consequently, we are careful to note that the observed interventions may have contributed to the high performance.

## CONCLUSION

The path to a resilient health system may be arduous and long, particularly for fragile countries. Therefore, it is important that any positive steps toward building resilient health systems should be acknowledged and nurtured. The silver lining of pandemics lies in the lessons learned. As a result of the COVID-19 pandemic, South Sudan and Sierra Leone have developed human resources and institutional capacity to respond to health emergencies while maintaining routine vaccination services and a near-real-time vaccination tracking dashboard. Both nations should invest adequate additional resources in the health sector to maintain these capabilities. In accordance with the Abuja Declaration, the governments should increase the current investment to at least 15% of the overall government budget.

## Supplementary Material

GHSP-D-23-00180-supplement2.pdf

GHSP-D-23-00180-supplement1.pdf

GHSP-D-23-00180-supplement3.pdf

GHSP-23-00180-Mokaya-article-summary_Portuguese.pdf

GHSP-23-00180-Mokaya-article-summary_French.pdf

GHSP-23-00180-Mokaya-article-summary_English.pdf

## Notes

**Peer Reviewed**

**First published online:** November 30, 2023.
