# Supplementary material for: Lessons From the COVID-19 Pandemic Response Implementation: A Case Study of South Sudan and Sierra Leone
Source: Glob Health Sci Pract. 2024 Feb 20;12(Suppl 1):e2300180. doi: 10.9745/GHSP-D-23-00180 (PMC10948125; doi:10.9745/GHSP-D-23-00180)
Supplement: GHSP-D-23-00180-supplement2.pdf [file GHSP-D-23-00180-supplement2.pdf]

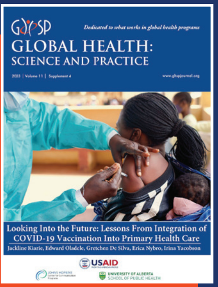

Leçons tirées de la mise en œuvre de la réponse à la pandémie de la COVID-19 : Une étude de cas du Sud-Soudan et de la Sierra Leone : Résumé de l’article

Evans Nyasimi Mokaya, Nathan Anyuon Atem, George Awzenio, Lawrence Mukombo, Tom Sesay, Desmond Maada Kangbai, Haurace Nyandemoh and Patience Musanhu

De quoi parle cet article ? Des acteurs clés du Sud-Soudan et de la Sierra Leone se penchent sur l’expérience acquise dans les deux pays lors de la mise en œuvre des activités de vaccination contre la COVID-19 dans leurs systèmes de santé fragile et identifient les pratiques qui ont eu un impact positif sur cette vaccination et l’immunisation de routine. Bien que la pandémie ait mis à rude épreuve les systèmes de santé des deux pays, les enseignements tirés de la riposte constituent une base pour l’intégration de la vaccination contre la COVID-19 dans les services de soins de santé primaires.

Quels étaient les résultats ? Plusieurs bonnes pratiques ont été identifiées dans chaque pays pour soutenir à la fois la vaccination contre la COVID-19 et la vaccination de routine.

Au Sud-Soudan :

- La formation et le recrutement de vaccinateurs, ainsi que le changement de politique visant à autoriser les vaccinateurs non infirmiers, ont permis de pallier la pénurie de personnel de santé et d’atteindre les objectifs de vaccination contre la COVID-19 tout en maintenant la couverture vaccinale de routine.
- L’intégration de la vaccination contre la COVID-19 à la vaccination de routine des enfants dans les cliniques mobiles et les services de

Points de vue des auteurs

Des auteurs des ministères de la santé de la Sierra Leone et du Sud-Soudan, du bureau national de l’OMS au Sud-Soudan et de Gavi, l’Alliance du Vaccin à Genève, en Suisse, ont collaboré pour la réalisation de cet article.

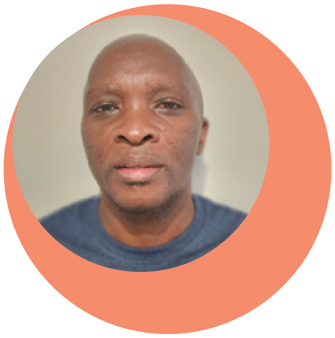

« Les leçons importantes qui guident le programme d’intégration dans les deux pays comprennent le renforcement de la confiance de la communauté dans les services de santé, la réduction des pénuries de vaccinateurs, l’intégration de programmes de formation et d’outils de supervision de soutien. »

- Dr. Evans Nyasimi Mokaya, Agent principal de programme, Ministère de la santé, Sud-Soudan

Lisez gratuitement l’article original publié dans Global Health Science and Practice à l’adresse suivante : https://doi.org/10.9745/GHSP-D-23-00180

proximité a permis d’accroître la vaccination systématique.

- L’élaboration d’un tableau de bord des vaccins contre la COVID-19 en temps quasi réel a contribué à une prise de décision rapide grâce aux données sur la couverture vaccinale dans l’ensemble du pays et a depuis été appliquée à la création d’un tableau de bord des vaccinations de routine.

En Sierra Leone

- La publicité faite autour de la vaccination du président et d’autres dirigeants politiques de haut niveau a permis de lutter contre les réticences et les préjugés généralisés à l’égard du vaccin contre la COVID-19.

Sierra Leone: couverture en pentavalent 3e dose, 2019-2022

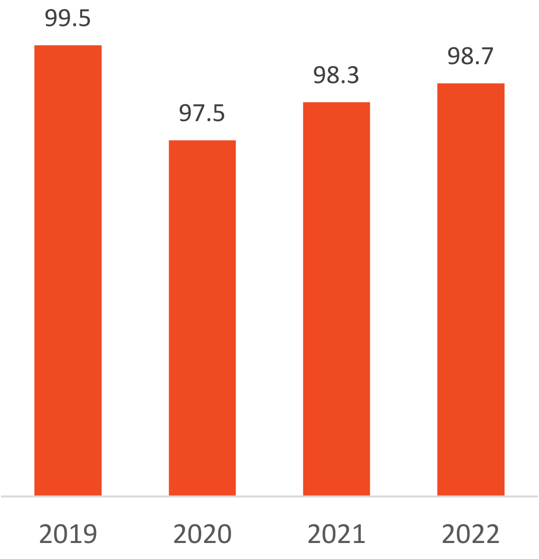

- L’intégration des vaccinations infantiles avec la vaccination contre la COVID-19 a permis d’inverser la tendance à la baisse des vaccinations infantiles observée au début de la pandémie.
- La création d’une base de données de vaccination basée sur les cas, intégrée à la plateforme DHIS2, a permis de collecter les données de vaccination contre la COVID-19 et d’envoyer des messages aux personnes vaccinées concernant leur prochaine dose.

Que signifient ces résultats ? Même dans les environnements fragiles, les services de vaccination de routine peuvent être maintenus pendant une pandémie. Les leçons tirées de la pandémie de COVID-19 en Sierra Leone et au Sud-Soudan devraient être appliquées aux futures urgences sanitaires dans les environnements fragiles. Les décideurs politiques et les autres parties prenantes devraient continuer à apporter un soutien financier et technique pour maintenir les innovations liées à la COVID-19 en matière de gestion des données, de formation et de fidélisation du personnel de santé supplémentaire.

Pourquoi cette étude a-t-elle été réalisée ? Avant la pandémie de COVID-19, le Sud-Soudan et la Sierra Leone ont connu des années de conflit, d'épidémies et d'autres difficultés, ce qui a affaibli les systèmes de santé, qui souffraient d'un manque de ressources humaines, de fournitures médicales et d'infrastructures adéquates. Ces systèmes de santé ont été mis à rude épreuve par la pandémie de la COVID-19. Peu de choses ont été écrites sur les efforts de réponse à la pandémie dans des contextes fragiles tels que le Sud-Soudan et la Sierra Leone. La compréhension des succès et des défis des activités de la COVID-19 au Sud-Soudan et en Sierra Leone fournit une feuille de route des meilleures pratiques pour les futures urgences dans des contextes similaires.

Quand et où cette étude a-t-elle été réalisée ? L'analyse a porté sur les activités de mise en œuvre de la vaccination contre la COVID-19 aux niveaux national et infranational au Sud-Soudan et en Sierra Leone entre 2020 et 2022.

USAID FROM THE AMERICAN PEOPLE

Knowledge SUCCESS

Ce guide a été réalisé grâce au soutien du peuple américain par l'intermédiaire de l'Agence américaine pour le développement international dans le cadre du projet Knowledge SUCCESS (Strengthening Use, Capacity, Collaboration, Exchange, Synthesis, and Sharing, accord de coopération n° 7200AA19CA00001 avec Johns Hopkins University. Knowledge SUCCESS est soutenu par le Bureau de la santé mondiale de l'USAID, le Bureau de la population et de la santé reproductive, et dirigé par le John Hopkins Centre for Communications Programs (CCP) en partenariat avec Amref Health Africa, le Centre d'économie comportementale de Busara et « FHI 360 ». Les informations fournies dans cette ressource relèvent de la seule responsabilité de Knowledge SUCCESS et ne reflètent pas nécessairement les opinions de l'USAID, du gouvernement américain ou de Johns Hopkins University.
