# Supplementary material for: Lessons From the COVID-19 Pandemic Response Implementation: A Case Study of South Sudan and Sierra Leone
Source: Glob Health Sci Pract. 2024 Feb 20;12(Suppl 1):e2300180. doi: 10.9745/GHSP-D-23-00180 (PMC10948125; doi:10.9745/GHSP-D-23-00180)
Supplement: GHSP-D-23-00180-supplement1.pdf [file GHSP-D-23-00180-supplement1.pdf]

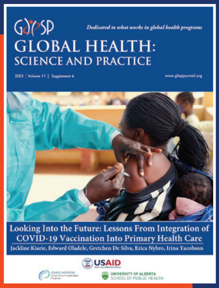

Lessons From the COVID-19 Pandemic Response Implementation: A Case Study of South Sudan and Sierra Leone: Article Summary

Evans Nyasimi Mokaya, Nathan Anyuon Atem, George Awzenio, Lawrence Mukombo, Tom Sesay, Desmond Maada Kangbai, Haurace Nyandemoh and Patience Musanhu

What is this article about? Key stakeholders in South Sudan and Sierra Leone reflect on experiences in both countries with the implementation of COVID-19 vaccination activities in their fragile health systems and identify practices that had a positive impact on COVID-19 vaccination and routine immunization. Although the pandemic further strained the health systems in both countries, lessons learned from the response provide a foundation for the integration of COVID-19 vaccination into primary health care services.

What were the results? Several best practices were identified in each country that supported both COVID-19 vaccination and routine immunization.

In South Sudan:

- Training and recruiting vaccinators and changing policy to allow non-nurse vaccinators alleviated health workforce shortages and helped achieve COVID-19 vaccination goals while maintaining routine immunization coverage.
- Integrating COVID-19 vaccination with routine childhood vaccination at mobile clinics and outreach services increased routine vaccination.
- Developing a near-real-time COVID-19 Vaccine Dashboard helped support rapid decision-making by providing data on vaccine coverage across the country and has since been applied to the creation of a routine immunization dashboard.

Insights from the Authors

Authors from the Sierra Leone and South Sudan Ministries of Health, as well as the South Sudan WHO Country Office, and Gavi, The Vaccine Alliance in Geneva, Switzerland collaborated on this article.

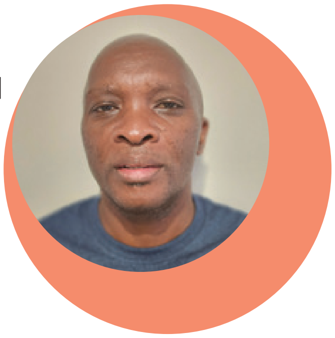

"Important lessons that are driving the integration agenda in both countries include building community trust in health facility services, alleviating shortages of vaccinators, integrating training packages, and supportive supervision tools."

- Dr. Evans Nyasimi Mokaya, Senior Program Coordinator, Ministry of Health, South Sudan

Read the original article published in Global Health Science and Practice for free at:
https://doi.org/10.9745/GHSP-D-23-00180

In Sierra Leone:

- Publicizing the vaccination of the President and other high-level political leaders helped to address widespread vaccine hesitancy and misconceptions about the COVID-19 vaccine.
- Integrating childhood immunizations with COVID-19 vaccination helped to reverse the decrease in childhood immunizations experienced early in the pandemic (see figure below).
- Creating a case-based vaccination database that was integrated with the DHIS2 platform collected COVID-19 vaccination data and sent messages to vaccine recipients about their next dose.

Sierra Leone: 3rd Dose Pentavalent Coverage, 2019-2022

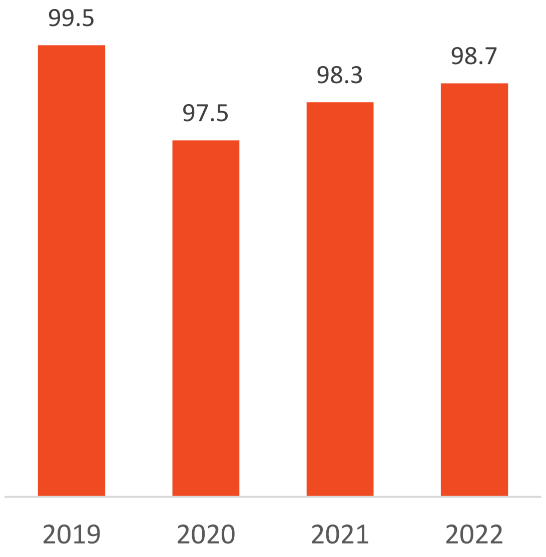

What do these results mean? Even in fragile settings, routine immunization services can be maintained during a pandemic. Lessons learned from the COVID-19 pandemic in Sierra Leone and South Sudan should be applied to future health emergencies in fragile settings. Policymakers and other stakeholders should continue to provide financial and technical support to maintain the COVID-19 innovations in data management and training and retention of the additional health workforce.

Why was this study done? Before the COVID-19 pandemic, both South Sudan and Sierra Leone experienced years of conflict, disease outbreaks, and other challenges, resulting in weak health systems that lacked human resources, medical supplies, and adequate infrastructure. These health systems were further strained by the COVID-19 pandemic. Little has been written about pandemic response efforts in fragile settings such as South Sudan and Sierra Leone. Understanding the successes and challenges of COVID-19 activities in South Sudan and Sierra Leone provides a road map of best practices for future emergencies in similar settings.

When and where was this study done? The analysis reviewed COVID-19 vaccination implementation activities at national and subnational levels in South Sudan and Sierra Leone from 2020 to 2022.

USAID FROM THE AMERICAN PEOPLE · Knowledge SUCCESS

This summary brief is made possible by the support of the American People through the U.S. Agency for International Development under the Knowledge SUCCESS (Strengthening Use, Capacity, Collaboration, Exchange, Synthesis, and Sharing) Project Cooperative Agreement No. 7200AA19CA00001 with the Johns Hopkins University.
Knowledge SUCCESS is supported by USAID's Bureau for Global Health, Office of Population and Reproductive Health and led by the Johns Hopkins Center for Communication Programs (CCP) in partnership with Amref Health Africa, The Busara Center for Behavioral Economics (Busara), and FHI 360. The information provided in this summary brief are the sole responsibility of Knowledge SUCCESS and does not necessarily reflect the views of USAID, the U.S. Government, or the Johns Hopkins University.
