# Supplementary material for: Lessons From the COVID-19 Pandemic Response Implementation: A Case Study of South Sudan and Sierra Leone
Source: Glob Health Sci Pract. 2024 Feb 20;12(Suppl 1):e2300180. doi: 10.9745/GHSP-D-23-00180 (PMC10948125; doi:10.9745/GHSP-D-23-00180)
Supplement: GHSP-D-23-00180-supplement3.pdf [file GHSP-D-23-00180-supplement3.pdf]

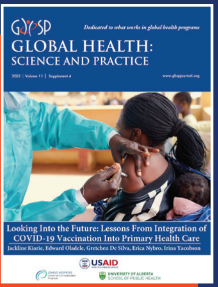

Lições da implementação da resposta à pandemia de COVID-19: um estudo de caso do Sudão do Sul e da Serra Leoa: resumo do artigo

Evans Nyasimi Mokaya, Nathan Anyuon Atem, George Awzenio, Lawrence Mukombo, Tom Sesay, Desmond Maada Kangbai, Haurace Nyandemoh and Patience Musanhu

De que trata este artigo? As principais partes interessadas no Sudão do Sul e na Serra Leoa refletem sobre as experiências em ambos os países com a implementação das atividades de vacinação contra a COVID-19 nos seus frágeis sistemas de saúde, e identificam práticas que tiveram um impacto positivo na vacinação contra a COVID-19 e na imunização de rotina. Embora a pandemia tenha sobrecarregado ainda mais os sistemas de saúde de ambos os países, os ensinamentos retirados da resposta constituem uma base para a integração da vacinação contra a COVID-19 nos serviços de cuidados de saúde primários.

Quais foram os resultados? Foram identificadas várias boas práticas em cada país que apoiaram tanto a vacinação contra a COVID-19 como a imunização de rotina.

No Sudão do Sul:

- Formação e recrutamento de vacinadores e alteração da política para permitir que os vacinadores não enfermeiros aliviem a escassez de mão de obra no setor da saúde e ajudem a atingir os objetivos de vacinação contra a COVID-19, mantendo simultaneamente a cobertura da imunização de rotina.
- A integração da vacinação contra a COVID-19 na vacinação infantil de rotina em clínicas móveis e serviços de proximidade aumentou a vacinação de rotina.

Perspetivas dos autores

Colaboraram neste artigo autores dos Ministérios da Saúde da Serra Leoa e do Sudão do Sul, bem como do Gabinete Nacional da OMS no Sudão do Sul e da Gavi, The Vaccine Alliance em Genebra, Suíça.

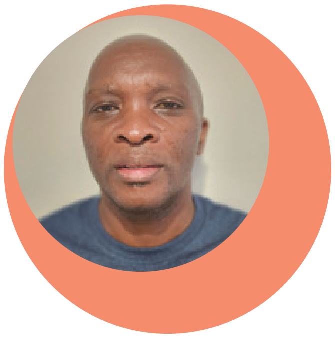

"As lições importantes que estão a impulsionar a agenda de integração em ambos os países incluem o reforço da confiança da comunidade nos serviços das unidades sanitárias, a redução da escassez de vacinadores e a integração de pacotes de formação e de ferramentas de supervisão de apoio."

- Dr. Evans Nyasimi Mokaya, Oficial Sénior de Programas do Ministério da Saúde, Sudão do Sul

Leia o artigo original publicado na Global Health Science and Practice gratuitamente em: https://doi.org/10.9745/GHSP-D-23-00180

- O desenvolvimento de um painel de controlo das vacinas contra a COVID-19 quase em tempo real ajudou a apoiar a tomada rápida de decisões, fornecendo dados sobre a cobertura vacinal em todo o país e, desde então, tem sido aplicado à criação de um painel de controlo das vacinas de rotina.

Na Serra Leoa

- A divulgação da vacinação do Presidente e de outros líderes políticos de alto nível ajudou a combater a hesitação generalizada e as ideias erradas sobre a vacina contra a COVID-19.

Serra Leoa: cobertura da 3ª dose de pentavalente, 2019-2022

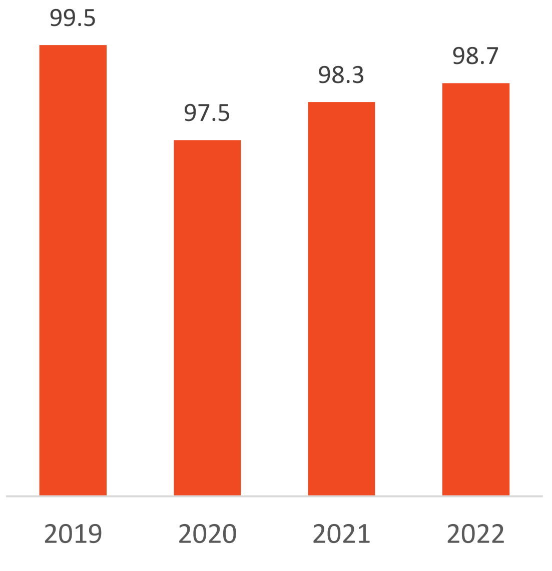

- A integração das imunizações infantis com a vacinação contra a COVID-19 ajudou a inverter a diminuição das imunizações infantis registada no início da pandemia.
- A criação de uma base de dados de vacinação baseada em casos, integrada na plataforma DHIS2, recolheu dados de vacinação contra a COVID-19 e enviou mensagens aos destinatários da vacina sobre a sua próxima dose.

O que significam estes resultados?

Mesmo em ambientes frágeis, os serviços de imunização de rotina podem ser mantidos durante uma pandemia. As lições aprendidas com a pandemia de COVID-19 na Serra Leoa e no Sudão do Sul devem ser aplicadas a futuras emergências de saúde em contextos frágeis. Os decisores políticos e outras partes interessadas devem continuar a prestar apoio financeiro e técnico para manter as inovações da COVID-19 na gestão de dados e na formação e retenção da força de trabalho adicional no setor da saúde.

Porque é que este estudo foi realizado?

Antes da pandemia de COVID-19, tanto o Sudão do Sul como a Serra Leoa viveram anos de conflito, surtos de doenças e outros desafios, o que resultou em sistemas de saúde fracos que careciam de recursos humanos, material médico e infraestruturas adequadas. Estes sistemas de saúde foram ainda mais afetados pela pandemia de COVID-19. Pouco se tem escrito sobre os esforços de resposta à pandemia em contextos frágeis como o do Sudão do Sul e da Serra Leoa. A compreensão dos êxitos e dos desafios das atividades da COVID-19 no Sudão do Sul e na Serra Leoa fornece um roteiro das melhores práticas para futuras emergências em contextos semelhantes.

Quando e onde foi efetuado este estudo? A análise reviu as atividades de implementação da vacinação contra a COVID-19 a nível nacional e subnacional no Sudão do Sul e na Serra Leoa de 2020 a 2022.

USAID FROM THE AMERICAN PEOPLE

Knowledge SUCCESS

Este guia resumido é possível graças ao apoio do povo americano através da U.S. Agency for International Development ao abrigo do Acordo de Cooperação do Projeto Knowledge SUCCESS (Strengthening Use, Capacity, Collaboration, Exchange, Synthesis, and Sharing) n.º 7200AA19CA00001 com a Universidade Johns Hopkins. O Knowledge SUCCESS é apoiado pelo Gabinete de Saúde Global, da População e Saúde Reprodutiva da USAID e dirigido pelo Johns Hopkins Center for Communication Programs (CCP) em parceria com a Amref Health Africa, The Busara Center for Behavioral Economics (Busara) e a FHI 360. As informações fornecidas neste guia resumido são da exclusiva responsabilidade da Knowledge SUCCESS e não refletem necessariamente as opiniões da USAID, do Governo dos EUA ou da Universidade Johns Hopkins.
